# 

**Published:** 1919-02-22

**Authors:** 


### London Inter-Collegiate Scholarships Board.

Thirty-one Scholarships and Exhibitions of an aggregate total value of about £3,075, open to men and women, and tenable in the Faculties of Arts, Science, Medical Sciences, and Engineering of University College, King's College, East London College, and Bedford College, will be offered for competition on Tuesday, May 13. Seventeen Medical Entrance Scholarships and Exhibitions of an aggregate total value of about £1,550, tenable in the Faculty of Medical Sciences of University College and King's College, and in the Medical Schools of Westminster Hospital, King's College Hospital, University College Hospital, the London (Royal Free Hospital) School of Medicine for Women, and the London Hospital, will be offered for competition on Tuesday, July 15. Particulars and entry forms may be obtained from the Secretary of the Board, Mr. S. C. Ranner, M.A., Medical School, King's College Hospital, Denmark Hill, London, S.E. 5.

### Londoners' Hospital Beds Threatened.

In the leading article under this heading in our issue of February 15, p. 417, the fifth line of the first column contains a serious error. Instead of "average available beds in 1,962 voluntary hospitals," it should be "in 116 voluntary hospitals."

### TUBERCULOSIS TOPICS.

### A Central Sanatorium.

At the last meeting of the Herts Insurance Committee the tuberculosis officer reported that the existing plan of sending sanatorium patients to three or four different institutions in the county was not a success, such patients frequently refusing to work, or even refusing to enter the institution at all. The vacant beds had to be paid for just the same, however. It was resolved, with unanimity, and almost with enthusiasm, to found a county sanatorium to accommodate all suitable cases of pulmonary tuberculosis, and a resolution was passed that representations in the above sense be made to the Hertfordshire County Council.

### Hull Tuberculosis Colony.

At the third annual meeting of the Hull Tuberculosis After-Care Committee, it was reported that at the tuberculosis colony at Walkington there were twelve colonists as inmates and one on the staff. The honorary secretary expressed the opinion that they were only at the commencement of their work at Walkington. Many new experiments would have to be undertaken in the near future, such as model workshops and houses, and the result might be a model village. All present were invited to visit the colony and see for themselves what they had every reason to believe would ultimately prove to be second to none in the Kingdom.

### A Disclaimer.

The Local Government Board has recently sent a communication to the Leeds National Health Insurance Committee taking notice of a statement appearing in the *Yorkshire Post* for August 17 last, and attributed to Dr. Woodcock, to the effect that the plan for the provision of extra beds had been fruitlessly submitted to a Government department; that the department had neglected to help in the matter, with the result of the plans not being passed. The Local Government Board denied, however, receiving the plans in question. It, moreover, stated that it would be glad to receive the Leeds City Council's observations on the matter, and to be informed when the plans and estimates, for which it originally asked as far back as last April, might be expected. As far as can be gathered from the ensuing discussion, this statement of the Local Government Board was justified, the Leeds Town Clerk stating that the City Engineer expected to get the plans finished in about a fortnight's time, he having been unable to proceed more quickly because of the lack of staff.

### Doctors' War Work in Hospitals.

### ORDER OF THE BRITISH EMPIRE.

THE KING has been pleased to give orders for the following appointments to the Order of the British Empire for valuable services rendered in or in connection with military hospitals, territorial hospitals, war hospitals, auxiliary and civil hospitals, command depôts, convalescent camps, or on other duties of a similar nature in the United Kingdom in connection with the Army during the war:

COMMANDERS (CIVIL DIVISION).

R. Alcock, M.B., F.R.C.S.; A. R. Anderson, F.R.C.S.; H. G. Frankling, M.R.C.S.; W. H. C. Greene, M.B., F.R.C.S.; R. G. Hogarth, F.R.C.S.; R. J. Howard, M.S., F.R.C.S.; W. J. Howarth, M.D., D.P.H.; Professor B. May, M.B., B.S., F.R.C.S.; W. C. Morton, M.D.; F. Nicholson, M.D.; A. J. Rice-Oxley, J.P., M.D., M.R.C.P.; T. Y. Simpson, M.D., M.S., F.R.C.S., L.R.C.P.

OFFICERS (CIVIL DIVISION).

R. Y. Aitken, M.D., F.R.C.S.; G. E. Genge-Andrews, M.B., B.S.; G. J. M. Atkinson, M.R.C.S., L.R.C.P.; I. Banks, M.D.; R. H. Barter, M.B., B.Ch.; W. C. Bentall, F.R.C.S., L.R.C.P.; E. J. Blackett, M.R.C.S., L.R.C.P.; J. Blomfield, M.D.; F. L. H. Brown, M.B., C.M.; Lieut.-Colonel W. H. Cadge, I.M.S.; J. Cashin, F.R.C.S.I., L.R.C.P.I.; J. J. Day, M.R.C.S., L.R.C.P., D.P.H.; J. Elliott, M.D., F.R.C.P.: H. B. Elton, M.B., B.C., M.R.C.S., L.R.C.P.; D. Ewart, M.D., Ch.B., F.R.C.S.; F. H. Fairweather, M.D., M.S.; H. D. Farnell, J.P., F.R.C.S.; E. T. Fison, M.D., D.P.H., F.R.C.S.; J. F. Fleming, M.B., Ch.B.; J. V. Fox, M.B., C.M.; J. D. Giles, M.D.; H. J. Godwin, M.B., B.S., F.R.C.S., L.R.C.P.; J. E. Gordon, M.R.C.S., L.R.C.P.; B. J. Guillemard, M.D.; J. A. Harrison, M.B., C.M.; J. H. Harvey, M.R.C.S., L.R.C.P.; E. D. H. Hawke, M.R.C.S., L.R.C.P.; A. W. Hunton, M.R.C.S., L.R.C.P.; Miss M. E. Jeremy, M.B., B.Ch.; J. R. Keele, M.R.C.S., L.R.C.P.; C. G. B. Kempe, M.D., B.S.; M. A. Key, M.B., B.Ch.; Miss M. F. Liston, M.B., Ch.B.; C. J. R. MacFadden, M.D., C.M.; J. Macintosh, M.D.; A. M. Mitchell, M.D., B.C., D.P.H.; H. C. Orrin, F.R.C.S.; R. C. Peacocke, M.D.; G. H. Percival, M.B., M.R.C.S.; C. J. Pinching, M.R.C.S., L.R.C.P.; A. Y. Pringle, M.R.C.S., L.R.C.P.; F. Radcliffe, M.B., Ch.B.; W. M. B. Rice, M.D., M.R.C.S.; K. Rogers, M.D.; Miss W. M. Ross, M.B., Ch.B.; S. H. Rouquette, M.B., M.Ch., F.R.C.S.; L. E. Shore, M.D., B.Ch.; E. W. Simmons, M.D.; C. D. Somers, M.B.; Miss F. A. Stoney, M.D., B.S.; J. W. Taylor, M.D., Ch.B.; B. B. T. Thorne, M.D., B.S.; J. W. T. Walker, M.B., C.M., F.R.C.S.; A. D. Webster, V.D., M.D., D.Sc., F.R.C.P.; C. G. R. Wood, F.R.C.S., L.R.C.P.; R. A. Worthington, M.B., B.C., F.R.C.S.

### Matrons' and Sisters' Appointments.

BOROUGH OF HASTINGS SANATORIUM, HASTINGS.—Miss Mary A. Holliday has been appointed matron. She was trained at the City of Westminster Infirmary, Fulham, and at the Western Fever Hospital. Miss Holliday has been sister at the Western Fever Hospital, sister and night superintendent at Shoreditch Infirmary, home sister and second assistant matron at the City of Westminster Infirmary, first assistant matron at the Township Infirmary, Leeds, and temporary matron at Folkestone Borough Sanatorium. Miss Holliday holds the certificate of the Central Midwives Board.

NORTH RIDING NURSING ASSOCIATION.—Miss Amy Bullock has been appointed lady superintendent and secretary, with supervision of the isolation hospitals under the Northallerton and Bedale District Councils. She was trained at the London Hospital, the Grosvenor Hospital for Women and Children, and at All Hallows' Hospital, Ditchingham. Miss Bullock has been temporary assistant matron at St. Mary's, Islington, Infirmary; home sister and housekeeper at the Royal Hants County Hospital, Winchester; lady superintendent and secretary of the Holt-Ockley Nursing Association; assistant superintendent of the Nurses' Institute, Canterbury; matron of the London Open-Air Sanatorium, Wokingham, the Children's Convalescent Home, Beaconsfield, the Infants' Hospital, Armley, Leeds, and of the War Hospital, Bradley Gate, Huddersfield.

STAMFORD, RUTLAND AND GENERAL INFIRMARY.—Miss Marianne Goodrich has been appointed matron. She was trained at the Royal Devon and Exeter Hospital; has been sister at the North-Eastern Fever Hospital, London, and at the Kent and Canterbury Hospital, Canterbury; night sister at the Royal Isle of Wight County Hospital, Ryde; out-patients' and casualty sister at the Royal Infirmary, Dundee; and lady superintendent and secretary of the North Riding Nursing Association, with charge of the isolation hospitals under the Northallerton and Bedale District Councils. Miss Goodrich has been a lecturer for the British Red Cross Society, and has done military work as a member of the Territorial Force Nursing Service.

CHARING CROSS HOSPITAL.—Miss Mary Cocrane has been appointed assistant matron, Miss Keyes Wells night superintendent, Miss Frow night sister, Miss Reay ward sister, and Miss Mallinson sister of the out-patient department. All were trained at the above hospital, where Miss Cocrane has been staff nurse and night superintendent, Miss Wells staff nurse and ward sister, and Miss Frow staff nurse. Miss E. H. Gilbert has been appointed sister tutor. She was trained at St. Bartholomew's Hospital; has been ward, night home sister and assistant matron at the Birmingham General Hospital; and sister in the Territorial Force Nursing Service.

### Manager's Notices.

**Now Ready, "The Hospital" Index to Vol. LXIV., April to September 1918. Price 6½d. per copy post free.**

**All letters on business matters must be addressed to The Manager, "The Hospital," 28 and 29 Southampton Street, London, W.C. 2.**

RATES OF SUBSCRIPTION (payable in advance).

UNITED KINGDOM.

| | | |
|---|---|---|
| Three Months | (including Postage) | 2s. 9d. |
| Six Months | do. | 5s. 5d. |
| Twelve Months | do. | 10s. 10d. |

FOREIGN AND COLONIAL.

| | | |
|---|---|---|
| Three Months | (including Postage) | 3s. 3d. |
| Six Months | do. | 6s. 6d |
| Twelve Months | do. | 13s. 0d. |

*SUBSCRIPTION FORM.*

*Please send me* THE HOSPITAL *for* .................. *months, commencing with your issue of* ........................., *for which please find enclosed* ........................

*Name* .........................................

*Address* .........................................

.........................................

*Date* .........................................

*To* ......................... *Newsagent.*

